# Seasonal Nile Crocodile Damage to Fishing Gear, Incident Reporting and Livelihood Responses in the Sudd Wetlands, South Sudan

**DOI:** 10.3390/ani16182826

**Published:** 2026-09-08

**Authors:** John Sebit Benansio, Gift Simon Damaya, Idika E. Okorie, Saralees Nadarajah

**Affiliations:** 1 AERD—Alliance for Environment and Rural Development, El Hikma Medical Centre Street, Gudele West, Block II, Juba P.O. Box 445, South Sudan; 2 Department of Fisheries Science, University of Juba, Juba P.O. Box 82, South Sudan; 3 Fisheries and Aquaculture Society of South Sudan, Ministry of Livestock and Fisheries, Juba P.O. Box 106, South Sudan; 4 Department of Wildlife Science, University of Juba, Juba P.O. Box 82, South Sudan; 5 Department of Mathematics, Khalifa University, Abu Dhabi P.O. Box 127788, United Arab Emirates; 6 Department of Mathematics, University of Manchester, Manchester M13 9PL, UK

**Keywords:** *Crocodylus niloticus*, human–crocodile conflict, artisanal fisheries, fishing nets, reporting pathways, Sudd Wetlands, South Sudan, ordinal regression, human–wildlife coexistence

## Abstract

Wetlands support fisheries and other livelihoods but can also bring people into close contact with crocodilians. Human–crocodile conflict has been documented across Africa, Asia, Australia and the Americas, yet indirect economic costs, including damage to fishing gear, remain less studied than attacks on people or livestock. We analysed questionnaire data from 379 respondents in purposively selected crocodile-exposed fishing communities in the southern Sudd Wetlands, South Sudan. The study quantified seasonal patterns of Nile crocodile (*Crocodylus niloticus*) damage to fishing gear, reporting pathways, conflict-resolution perceptions, responses to damaged gear and likely annual damaged-net categories. Reporting pathways were defined as the social and institutional channels through which respondents said gear-damage incidents were communicated; handling responses were defined as actions taken after gear was damaged. As expected from seasonal variation in floodplain use, the water level and fishing activity, reported damage was concentrated from July to December, with September the being most frequently selected main damage month (157 respondents; 41.4%). Quarterly ordinal responses differed among seasons (Friedman test: $\chi^{2}=721.54$, df = 3, p<0.001). Cumulative link models that included both quarters and the broader survey area showed the largest odds-ratio contrasts for July–September and October–December, whereas broader-area contrasts were smaller, and their confidence intervals overlapped one. Reporting was dominated by informal and community pathways: fishing-camp leadership, family members and other fishers each reached 100% agreement, whereas wildlife authorities received no affirmative reporting. Respondents also expressed little confidence in formal conflict resolution: 100% agreed that crocodile-related gear-damage conflicts were difficult to resolve, and 0% agreed that government would compensate for damaged gear. Damaged nets were mainly abandoned and replaced; repair was rare (1.6%). The most likely annual damaged-net category was 1–5 nets/year/participant (275 respondents; 72.6%). Because the survey was purposive, the results should be interpreted as patterns among sampled respondents and locations, not as prevalence estimates for all fishers in the Sudd Wetlands or South Sudan. The findings indicate that seasonal gear damage, weak formal reporting to wildlife institutions and limited institutional confidence should be central considerations in crocodile-conflict management.

## Simple Summary

Crocodiles and people often depend on the same wetland habitats. Around the world, human–crocodile conflict is usually discussed in relation to attacks on people or livestock, but crocodiles can also affect livelihoods by damaging fishing gear and reducing fishing opportunities. This study examined reported Nile crocodile damage to fishing gear among purposively selected fishing participants in the southern Sudd Wetlands, South Sudan. Damage was strongly seasonal, with the main reported months falling between July and October and September selected most often. Respondents mainly communicated incidents through local and informal channels, including other fishers, family members and fishing-camp leadership. Confidence in formal conflict-resolution or compensation systems was low, and damaged nets were usually abandoned and replaced rather than repaired. Because the survey was purposive, the findings should be interpreted as patterns among sampled crocodile-exposed communities rather than as estimates for all fishers in South Sudan. The results suggest that seasonal warnings, local reporting systems and practical support for documenting and responding to damaged gear should be considered in crocodile-conflict management.

## 1. Introduction

Wetlands are among the most productive ecosystems globally and support fisheries, grazing, transport, flood regulation, biodiversity conservation and household water needs [1]. For communities living within or beside large floodplain wetlands, these ecosystem services are also livelihood infrastructure: they provide fish, pasture, fertile soils and seasonal mobility. The same ecological features that make wetlands valuable to people also support large aquatic predators. Where human livelihoods and crocodilian habitat overlap, conflict can emerge through attacks on people, predation on livestock, depredation of captured fish, damage to fishing gear, fear, retaliatory killing and contested responsibilities for management [2,3,4,5].

Human–wildlife conflict is not only a problem of animal behaviour. It is a social–ecological process shaped by risk, livelihood dependence, local institutions, trust in authorities, cultural meaning and the perceived fairness of conservation costs [2,6,7,8,9]. This perspective is important for crocodilian conflict because crocodiles often occupy waters that local people use daily for fishing, livestock watering, transport, bathing and domestic activities. International studies show that human–crocodile interactions vary geographically and institutionally, including in India, Mexico, Australia and southern Africa [4,10,11,12,13]. These studies collectively show that conflict is influenced by both ecological seasonality and the ways people report, interpret and respond to encounters.

Most crocodile-conflict research has emphasized attacks on humans and livestock [4,14,15,16]. Less attention has been given to indirect but tangible livelihood costs, especially fishing-gear damage. For artisanal fishing households, nets are productive assets rather than incidental equipment. Damage to nets may reduce fishing time, force borrowing from colleagues, require replacement, increase debt or reduce household food and income. Gear damage may therefore contribute to negative attitudes toward crocodiles, even when no human injury occurs. Understanding this pathway is important because compensation, reporting systems and mitigation trials are often designed around dramatic attacks rather than recurring material losses.

The Nile crocodile (*Crocodylus niloticus*) is widespread in sub-Saharan Africa and is globally listed as Least Concern, although local populations can be affected by retaliatory killing, illegal harvest and habitat disturbance [17,18]. In South Sudan, the Sudd Wetlands are among the world’s largest freshwater wetland systems and are closely linked to White Nile hydrology, seasonal inundation and floodplain productivity [19,20,21,22]. The wetland supports fishing, agro-pastoralism and livestock production while also providing suitable crocodile habitat. Previous studies in the Sudd have documented Nile crocodile attacks and local attitudes toward crocodiles [23,24]; however, fishing-gear damage, reporting pathways and livelihood responses remain under-analysed.

This study examined crocodile-related fishing-gear damage among purposively sampled fishing participants in the southern Sudd Wetlands. We asked five linked questions: (1) how does reported fishing-gear damage vary seasonally; (2) which months are most often selected as the main period of damage; (3) through which reporting pathways are gear-damage incidents communicated; (4) how do respondents perceive conflict resolution and institutional support; and (5) what actions are taken after fishing gear is damaged, and what annual damaged-net categories are reported? We expected reported damage to increase during floodplain and late wet-season periods, when fishing activity and crocodile use of productive aquatic habitats are likely to overlap. We also expected reporting to be dominated by informal community channels because remote wetland settlements often have limited access to formal wildlife-management institutions.

## 2. Materials and Methods

### 2.1. Study Area

The study was conducted in the southern zone of the Sudd Wetlands, South Sudan, including Terekeka, Northern Terekeka, Gemeiza, Tombek and Mangalla in Central Equatoria State. South Sudan is a landlocked country in East–Central Africa, and the Sudd is a large White Nile wetland complex whose extent varies seasonally with hydrology and floodplain inundation [19,20,21,22]. The study settlements are located along the White Nile and associated wetland margins, where fishing, agro-pastoralism, livestock keeping and small-scale cultivation are important livelihoods.

**Figure 1:**
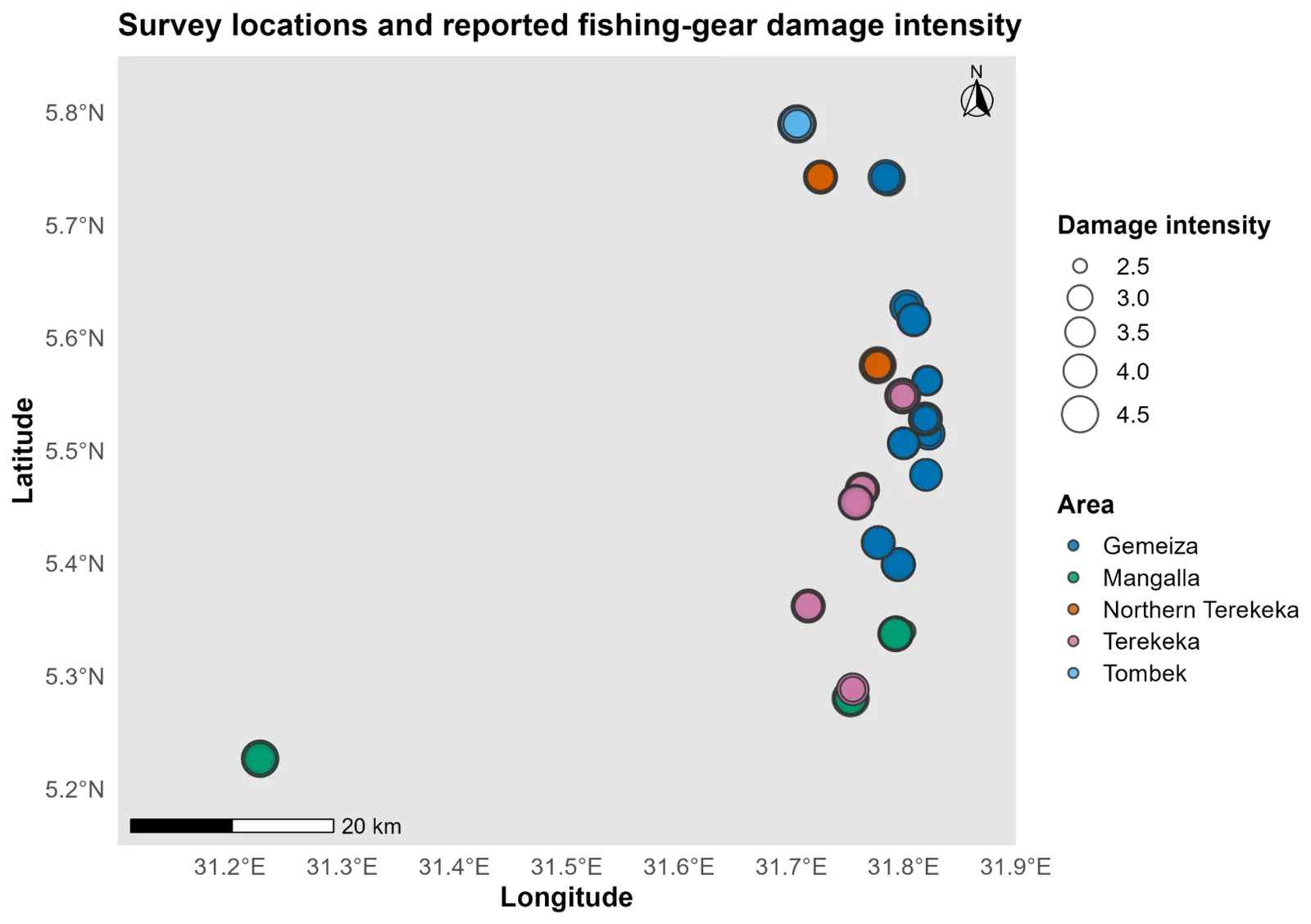
Survey locations and reported fishing-gear damage intensity in the southern Sudd Wetlands. Point size represents mean the quarterly damage intensity, and point colour represents the survey area. The map shows sampled locations only.

The Sudd provides extensive habitat for Nile crocodiles, including channels, lagoons, reed beds, papyrus stands and seasonally flooded margins. Local communities use these same waters for fishing, movement, livestock watering, bathing and other household activities. The major ethnic groups in the southern study zone include the Mundari and Bari communities. Dominant fishing gear in the study area includes stationary cotton gill nets and stationary nylon or monofilament gill nets. The mapped survey points used in this analysis extended approximately from 5.2 to 5.8° N and from 31.2 to 31.9° E (Figure 1). The map is intended to show sampled locations and reported damage intensity, not to infer unsampled risk across the whole Sudd Wetlands.

### 2.2. Survey Design, Sampling and Ethical Procedures

The interview survey was conducted on the first day of every month from July 2018 to June 2020 by a field team of three male researchers. The southern Sudd study areas were selected purposively because they were accessible; supported fishing activity; depended on wetland livelihoods and had frequent local reports of Nile crocodile occurrence, fishing-gear damage, crocodile attacks on humans and livestock, juvenile crocodile by-catch and crocodile sightings. This design was appropriate for documenting crocodile-related gear damage in exposed communities, but it does not support prevalence estimates for all fishers in the Sudd Wetlands or South Sudan.

Respondents were selected with the permission of fishing-village or fishing-camp heads and with the assistance of local wildlife officers. Eligibility criteria included being an adult resident of the study area, having local knowledge or experience of Nile crocodiles, participating in fishing or fishing-related wetland livelihoods, and willingness to participate. The sample therefore included fishers and part-time fishing participants from agro-pastoralist and pastoralist households. Throughout the manuscript, the term “participant” refers to this sampled group; where “fishers” is used, it refers to respondents involved in fishing or fishing-related activities rather than to a probability sample of all fishers in South Sudan.

The questionnaire was administered individually in local Arabic or Bari and typically lasted 25–30 min. Respondents were informed about the purpose of the study, participation was voluntary, verbal informed consent was obtained, and respondents could decline the interview or any question. Personal names were optional and are not reported. Women were under-represented because participation often required permission from male partners. The field record included 21 women and 357 men; one respondent in the analytical dataset had no usable gender code. Thus, women represented approximately 5.5% of the analytical sample. Female and male respondents were interviewed separately where possible to reduce sensitivity and social pressure.

The questionnaire was pre-tested before field deployment. Fifteen pilot interviews were conducted over 10 days before the main survey to assess interview duration, question clarity, response formats and whether the survey captured information meaningful to respondents. Ambiguous questions were revised before the main survey. The analysed questions, response formats and coding are summarized in Appendix A Table A1; the full questionnaire is submitted as Supplementary File S1.

### 2.3. Data Preparation and Response Variables

The cleaned analytical dataset contained 379 respondents from 27 villages or fishing locations across five broader areas: Terekeka (83 respondents; 21.9%), Tombek (81; 21.4%), Gemeiza (80; 21.1%), Mangalla (70; 18.5%) and Northern Terekeka (65; 17.2%). Location-level counts are reported in Supplementary Table S1.

Quarterly gear-damage frequency was recorded for January–March, April–June, July–September and October–December using ordered response categories. For analysis, responses were coded from 1 to 5, with larger values representing more frequent reported damage: 1 = never, 2 = rarely, 3 = occasionally, 4 = often and 5 = most of the time. The ordered nature of this response was retained in ordinal models.

The main damage-month variable recorded the month respondents selected as the main period of fishing-gear damage. Reporting pathways were treated as agreement responses for whether damage was reported through specific channels, including other fishers, family members, fishing-camp leadership, fisheries authorities, community leaders, research or academic institutions and wildlife authorities. Agreement with conflict-resolution statements and responses to damaged gear were coded by combining “agree” and “strongly agree” responses. Handling responses included repairing damaged nets, abandoning damaged nets, borrowing nets from colleagues and purchasing new nets. The annual damaged-net category was summarized as a likely mutually exclusive category per participant per year using the ordered categories of 1–5, 6–10, 11–15 and >20 nets/year/participant.

### 2.4. Statistical Analysis

The analysis was organized by research question rather than as a generic list of tests (Table 1). Descriptive statistics were used for respondent distribution, reporting pathways, conflict-resolution perceptions, handling responses and annual damaged-net categories. The Friedman test was used to compare repeated quarterly ordinal scores across respondents. The main damage-month distribution was compared against a uniform distribution using a chi-square goodness-of-fit test. Cochran’s Q test was used to compare repeated binary agreement indicators for handling responses.

Quarterly damage frequency was the primary regression response. Because each respondent provided four ordered quarterly responses, the main model class was a cumulative link mixed model. Candidate quarterly models included quarter, area and available respondent-level covariates, and random intercepts for respondent and/or village/fishing location were evaluated to address repeated observations and possible clustering among respondents from the same location. A fixed-effect cumulative logit model with the same main predictors was fitted to report interpretable odds ratios. Candidate models were compared using Akaike’s information criterion (AIC), but AIC comparisons were made only among models fitted to the same response variable.

For formal reporting, a binary indicator was created for agreement with reporting to fisheries or wildlife authorities and analysed using logistic regression when both outcome classes were available. For handling responses, binary logistic regression was considered separately for each handling action. Repair, abandonment and purchase were not interpreted as regression outcomes because repair had six affirmative events (1.6%) and abandonment/purchase lacked usable variation. Borrowing was analysed with binary logistic regression. The collapsed annual damaged-net category was analysed using proportional-odds ordinal logistic regression. A midpoint approximation of annual damaged-net counts was analysed only as a supplementary sensitivity analysis using Poisson and negative-binomial generalized linear models; the negative-binomial model was considered when Poisson overdispersion was present. Pairwise associations among categorical predictors were summarized using Cramér’s *V* as a descriptive effect-size measure for association between categorical variables. Cramér’s *V* was not interpreted as a linear correlation coefficient. The largest observed association was between age group and origin status (Cramér’s V=0.31), and no predictor pair showed a sufficiently strong categorical association to require exclusion from the candidate models. R version 4.5.0 was used for all analyses [25]. Data import and wrangling used readr, dplyr, tidyr, stringr, forcats and purrr [26,27,28,29,30,31]. Graphics used ggplot2, scales, viridis, patchwork and ragg [32,33,34,35,36]. Model tidying used broom [37]. Ordinal, logistic and count models used ordinal and MASS [38,39]; the specific modelling references were cited in relation to the models actually fitted: cumulative link models for ordered quarterly and annual responses, logistic regression for binary reporting or handling outcomes, Poisson and negative-binomial generalized linear models for the supplementary approximate count analysis, and AIC for comparing candidate models within the same response variable. Models fitted to different outcomes were not compared using AIC. [40,41,42,43,44,45]. Spatial mapping used sf, rnaturalearth, rnaturalearthdata and ggspatial [46,47,48,49].

### 2.5. Analytical Expressions of Fitted Regression Models

Let i=1,…,n index respondents, j=1,…,4 index quarters, *ℓ* index village or fishing location, and *c* index ordered response thresholds. Let $Y_{ij}$ denote the quarterly ordered damage-frequency response, $q_{j}$ denote quarter, $a_{i}$ denote area, $t_{i}$ denote survey year and $x_{i}$ denote a vector of respondent-level covariates. The cumulative link mixed model was

(1) $$\mathrm{logit}\left\{\mathrm{Pr}\left(Y_{ij}\leq c|b_{i},u_{l[i]}\right)\right\}=\theta_{c}-\left(\beta_{q}^{⊤}q_{j}+\beta_{a}^{⊤}a_{i}+\beta_{t}^{⊤}t_{i}+\gamma^{⊤}x_{i}+b_{i}+u_{l[i]}\right),$$

where $b_{i}∼N\left(0,\sigma_{b}^{2}\right)$ is a respondent random intercept and $u_{l}∼N\left(0,\sigma_{u}^{2}\right)$ is a location random intercept. Candidate models included subsets of these fixed effects and random effects. The fixed-effect cumulative logit model used for interpretable odds ratios was

(2) $$\mathrm{logit}\left\{\mathrm{Pr}\left(Y_{ij}\leq c\right)\right\}=\theta_{c}-\left(\beta_{q}^{⊤}q_{j}+\beta_{a}^{⊤}a_{i}\right).$$

Because larger observed categories represented more frequent damage, odds ratios greater than one indicate higher odds of being in a more frequent damage category.

For a binary outcome ($Z_{i}$), such as formal reporting or borrowing nets from colleagues, logistic regression was

(3) $$Z_{i}∼\mathrm{Bernoulli}\left(p_{i}\right),\;\mathrm{logit}\left(p_{i}\right)=\beta_{0}+\beta_{a}^{⊤}a_{i}+\beta_{t}^{⊤}t_{i}+\gamma^{⊤}x_{i}.$$

For the ordered annual damaged-net category ($L_{i}$), the proportional-odds model was

(4) $$\mathrm{logit}\left\{\mathrm{Pr}\left(L_{i}\leq c\right)\right\}=\theta_{c}^{L}-\left(\beta_{0}+\beta_{a}^{⊤}a_{i}+\beta_{t}^{⊤}t_{i}+\gamma^{⊤}x_{i}\right).$$

For the supplementary approximate annual count ($C_{i}$), Poisson and negative-binomial models used a log link, i.e.,

(5) $$\begin{array}{cc} \; & C_{i}∼\mathrm{Poisson}\left(\mu_{i}\right),\;\log\left(\mu_{i}\right)=\eta_{i},\;\;\;\; \end{array}$$

(6) $$\begin{array}{cc} \; & \;\;\;\;\;C_{i}∼\mathrm{NB}\left(\mu_{i},\kappa \right),\;\log\left(\mu_{i}\right)=\eta_{i},\;\mathrm{Var}\left(C_{i}\right)=\mu_{i}+\mu_{i}^{2}/\kappa . \end{array}$$

For each outcome-specific candidate set, model support was compared using

(7) $${\mathrm{AIC}}_{m}=-2\log\left(L_{m}\right)+2K_{m},$$

where $L_{m}$ is the maximized likelihood and $K_{m}$ is the number of estimated parameters. AIC values were not compared across different response variables.

## 3. Results

### 3.1. Sample Profile and Survey Locations

The final analytical dataset contained 379 respondents from 27 villages or fishing locations across five broader areas (Table 2). Respondents were distributed across Terekeka (83; 21.9%), Tombek (81; 21.4%), Gemeiza (80; 21.1%), Mangalla (70; 18.5%) and Northern Terekeka (65; 17.2%). Survey years spanned 2018–2020. The field gender record contained 357 men, 21 women and one respondent without a usable gender code. Age-group information showed 103 respondents aged 19–29 years, 189 aged 30–49 years, 76 aged 50–69 years and 10 aged 70 years or older. The largest single location was Tombek (81 respondents); the remaining locations had smaller sample sizes and are reported in Supplementary Table S1.

### 3.2. Seasonality of Fishing-Gear Damage

**Figure 2:**
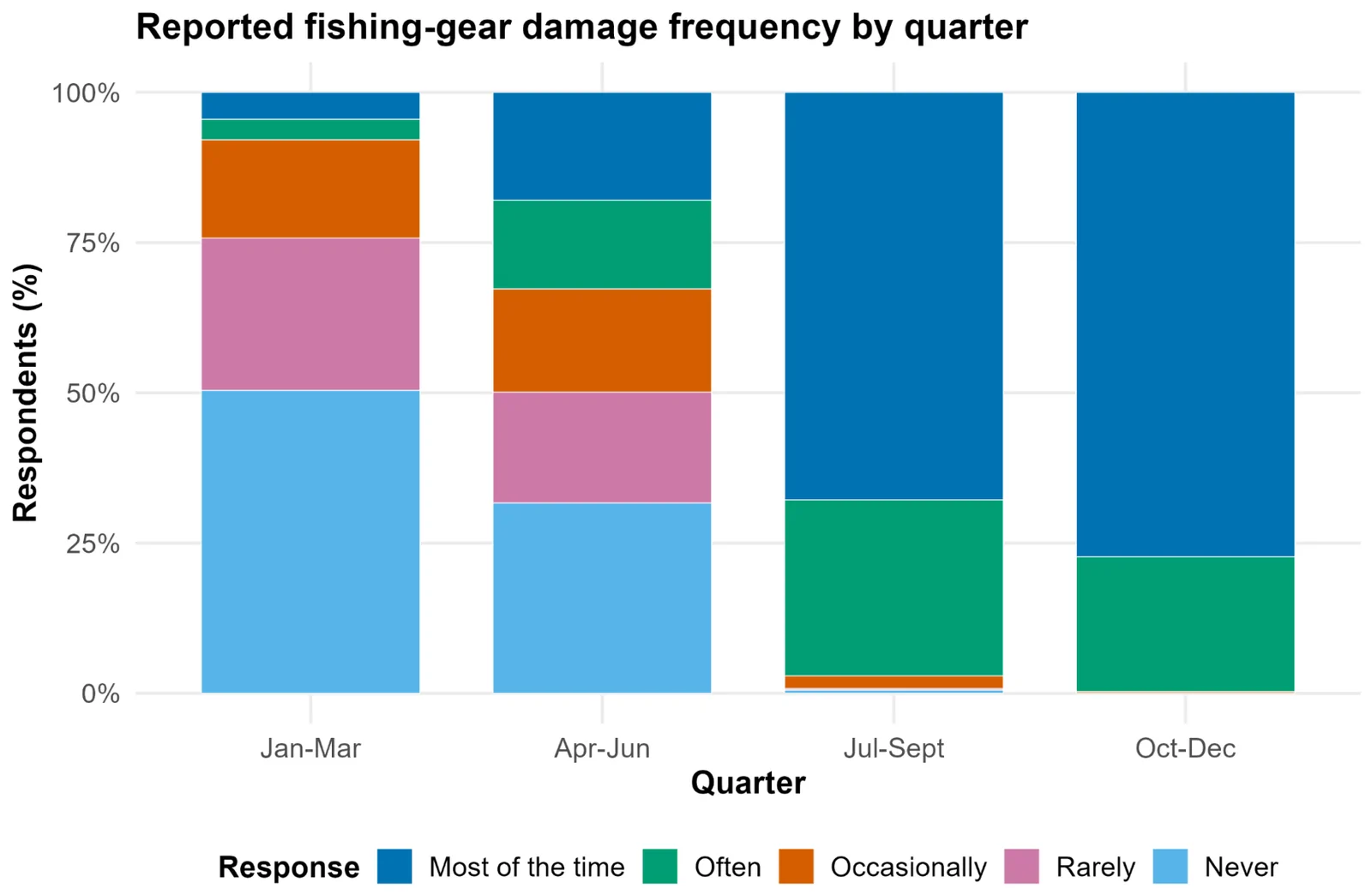
Reported fishing-gear damage frequency by quarter. Bars sum to 100% within each quarter.

Reported fishing-gear damage frequency varied among quarters (Figure 2). Responses of “never” and “rarely” were concentrated in January–March and April–June, whereas “often” and “most of the time” dominated July–September and October–December. Mean intensity scores increased from January–March (mean = 1.86, SE = 0.06) and April–June (mean = 2.69, SE = 0.08) to July–September (mean = 4.64, SE = 0.03) and October–December (mean = 4.77, SE = 0.02). The Friedman test showed difference among quarters ($\chi^{2}=721.54$, df = 3, p<0.001).

The quarterly cumulative link mixed-model comparison supported models containing quarter and area (Table 3). Models with a location random intercept and a respondent random intercept had nearly identical AIC values, indicating that accounting for clustering did not alter the dominant seasonal result. The fixed-effect cumulative logit model used for odds-ratio reporting showed that, relative to April–June, odds of being in a higher damage-frequency category were lower in January–March (OR = 0.33, 95% CI = 0.25–0.43) and much higher in July–September (OR = 24.51, 95% CI = 17.55–34.23) and October–December (OR = 40.22, 95% CI = 28.14–57.48). Area effects were smaller, and confidence intervals overlapped one.

**Table 3:** Summary of fitted model families and interpretation status. AIC comparisons were made only within the same response variable.

Outcome	Candidate Models Fitted	Best-Supported Model/Status	Main-Text Use
Quarterly damage intensity, mixed ordinal	Random-intercept null; quarter; quarter + area; full; respondent and location random effects evaluated	Quarter + area + location random intercept; nearly identical to respondent random-intercept model	Primary seasonal model
Quarterly damage intensity, fixed ordinal	Null; quarter; quarter + area; full	Quarter + area	Odds ratios reported
Formal reporting, binary logistic	Null; area/year; demographic; full	Null	Descriptive result emphasized
Borrowing nets, binary logistic	Null; area/year; demographic; full	Area/year	Supplementary interpretation only
Repairing nets	Considered but not fitted	Rare events	Descriptive only
Abandoning damaged nets	Considered but not fitted	No usable outcome variation	Descriptive only
Purchasing new nets	Considered but not fitted	No usable outcome variation	Descriptive only
Annual damaged-net category, ordinal	Null; area/year; demographic; full	Full; year effects most interpretable	Secondary model
Annual damaged-net count	Poisson and negative-binomial null, area/year, demographic and full models	Negative-binomial full	Supplementary sensitivity analysis

### 3.3. Main Months of Gear Damage

**Figure 3:**
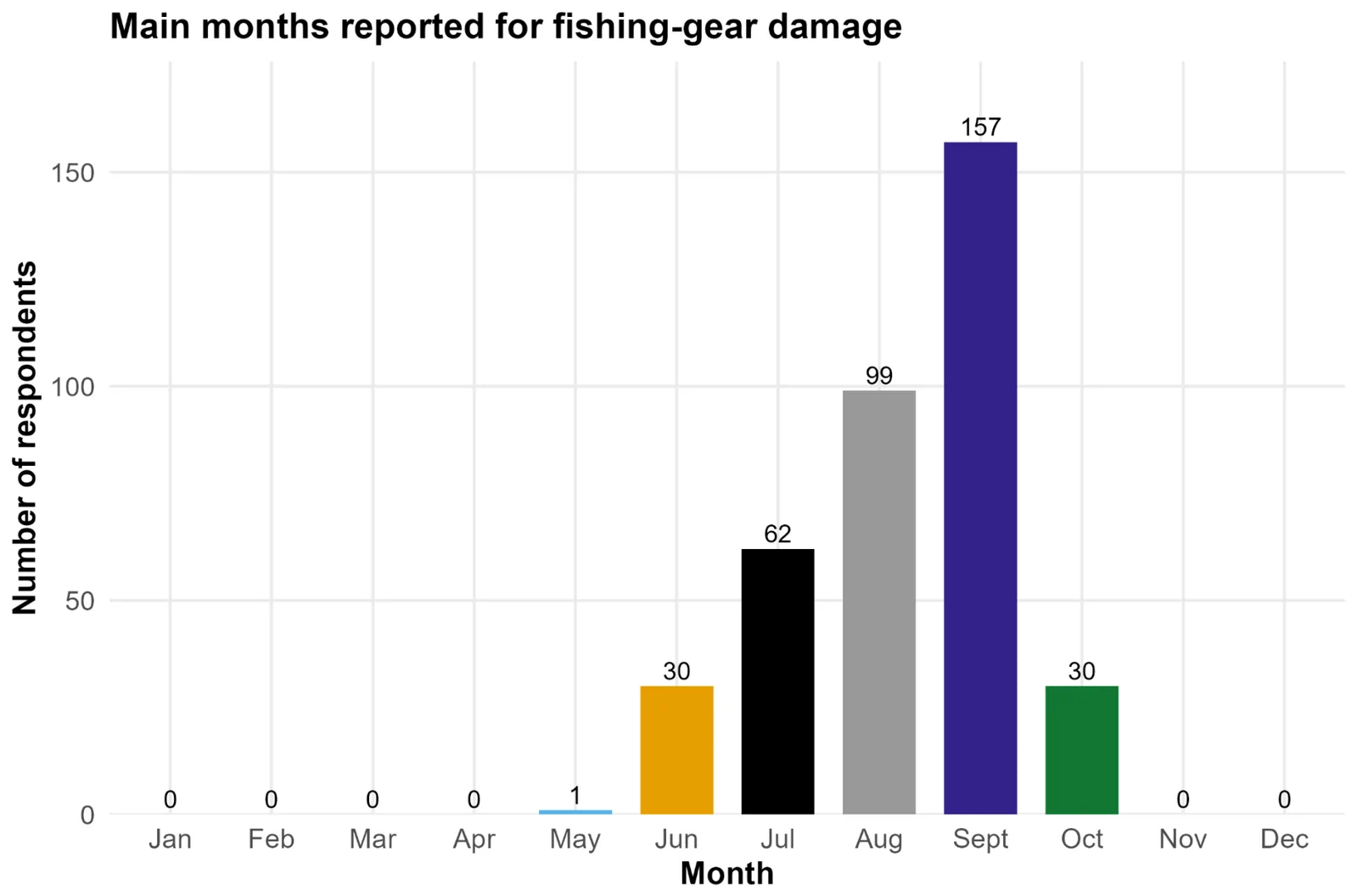
Main months reported for fishing-gear damage caused by Nile crocodiles.

The selected main damage month was concentrated in the late wet-season period (Figure 3). September was selected by 157 respondents (41.4%), followed by August (99; 26.1%), July (62; 16.4%), June (30; 7.9%), October (30; 7.9%) and May (1; 0.3%). No respondents selected January–April, November or December. The monthly distribution differed from uniformity ($\chi^{2}=890.50$, df = 11, p<0.001).

The quarterly and monthly variables measured different aspects of seasonality. The quarterly question asked respondents to score damage frequency within each three-month period, whereas the monthly question asked respondents to select the main month of damage. Therefore, September could be the dominant single month, while October–December retained a high quarterly frequency score. Cross-tabulations between selected main month and quarterly responses were used as a data-checking output and are reported in Supplementary Table S2.

### 3.4. Reporting Pathways and Conflict-Resolution Perceptions

**Figure 4:**
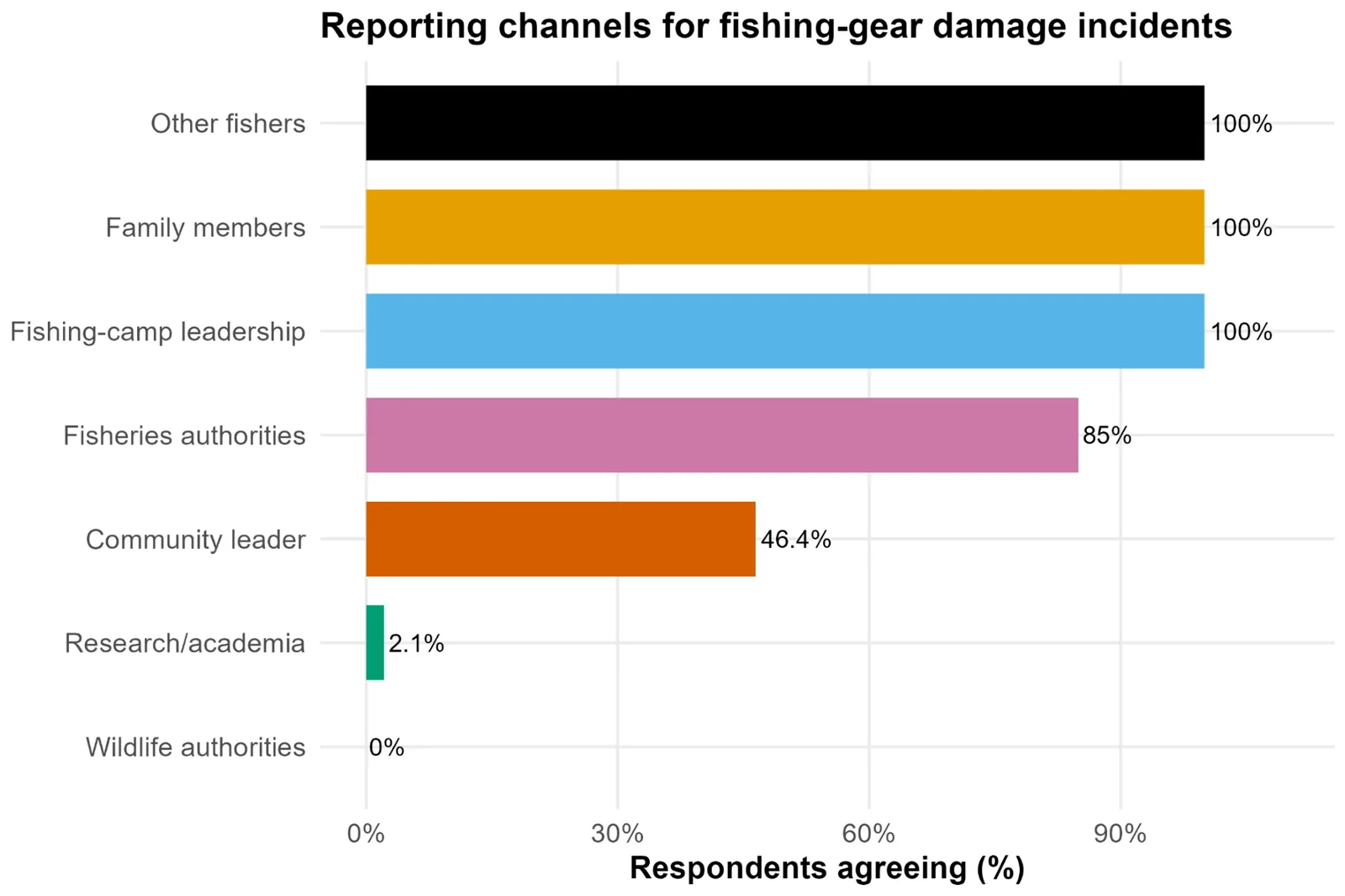
Reporting channels for fishing-gear damage incidents. Agreement includes “agree” and “strongly agree” responses.

Reporting pathways were dominated by informal or community channels (Figure 4). All respondents agreed that gear-damage incidents were reported to other fishers, family members and fishing-camp leadership. Agreement was also high for reporting to fisheries authorities (322/379; 85.0%). Fewer respondents agreed that incidents were reported to community leaders (176/379; 46.4%), research or academic institutions (8/379; 2.1%) or wildlife authorities (0/379; 0%). The formal-reporting logistic regression candidate set favoured the null model (see Supplementary Table S3), so measured covariates were not interpreted as meaningful predictors of formal reporting.

**Figure 5:**
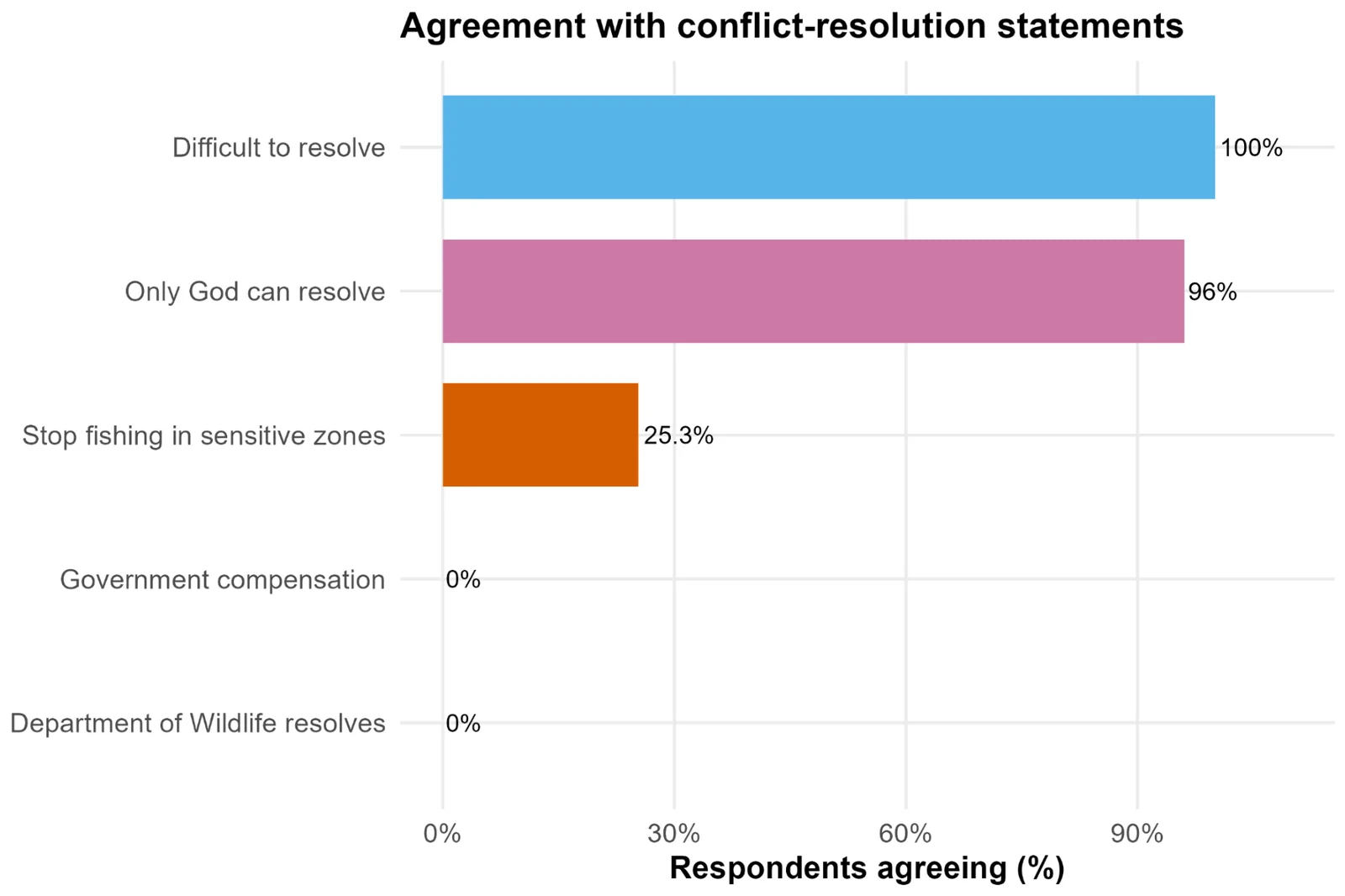
Agreement with conflict-resolution statements. Agreement includes “agree” and “strongly agree” responses.

Conflict-resolution perceptions showed low confidence in formal institutional solutions (Figure 5). All respondents agreed that crocodile-related gear-damage conflicts were difficult to resolve (379/379; 100%). Most respondents agreed that only God could resolve the conflict (364/379; 96.0%). Agreement was lower for stopping fishing in crocodile-sensitive zones (96/379; 25.3%). No respondent agreed that the Department of Wildlife could resolve the issue or that government would compensate for damaged fishing gear.

### 3.5. Responses to Damaged Fishing Gear

**Figure 6:**
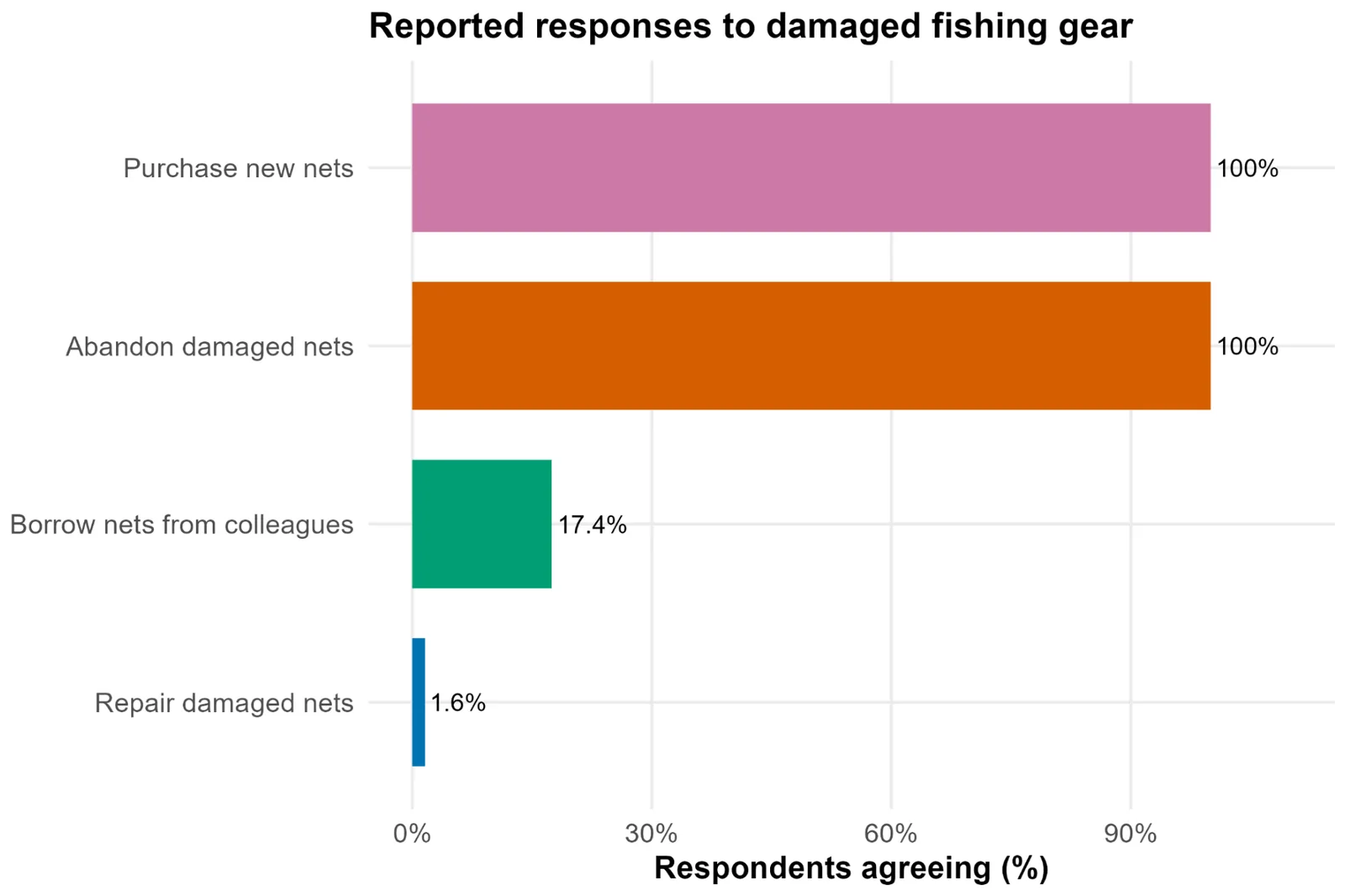
Reported responses to damaged fishing gear. Agreement includes “agree” and “strongly agree” responses.

Reported responses to damaged fishing gear were dominated by abandonment and replacement (Figure 6). All respondents agreed that damaged nets were abandoned and that new nets were purchased. Borrowing nets from colleagues was reported by 66 respondents (17.4%), while repair was reported by only six respondents (1.6%). Cochran’s Q test showed that agreement differed strongly among the four handling responses (Q=992.65, df = 3, p<0.001). Because repair had six affirmative responses (1.6%) and abandonment/purchase lacked usable variation, only borrowing was evaluated with binary logistic regression. The borrowing model is reported as Supplementary Table S4) because the behavioural interpretation of area and year effects is limited.

### 3.6. Likely Annual Damaged-Net Category

**Figure 7:**
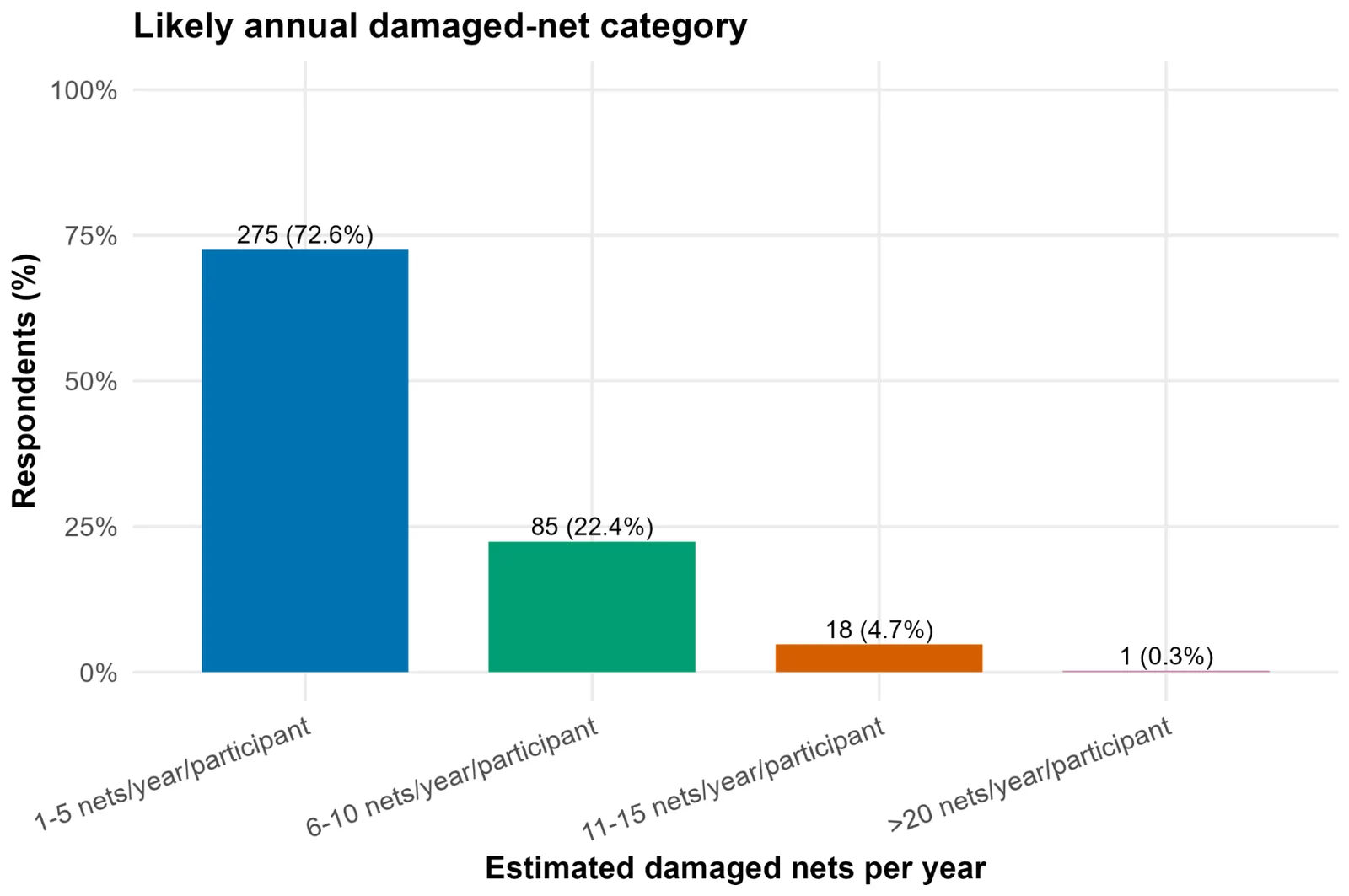
Likely annual damaged-net category per participant. Categories are mutually exclusive.

The likely annual damaged-net category was concentrated in the lower categories (Figure 7). Most respondents were assigned to 1–5 nets/year/participant (275 respondents; 72.6%), followed by 6–10 nets/year/participant (85; 22.4%), 11–15 nets/year/participant (18; 4.7%) and >20 nets/year/participant (1; 0.3%). The ordinal model for the collapsed annual damaged-net category selected the full model by AIC, although year effects were the main interpretable pattern (see Supplementary Table S5). Relative to 2018, odds of being in a higher damaged-net category were lower in 2019 (OR = 0.31, 95% CI = 0.17–0.56) and 2020 (OR = 0.21, 95% CI = 0.11–0.37). University-level education was associated with higher odds of a higher annual damaged-net category (OR = 4.86, 95% CI = 1.63–14.47), but this association should be interpreted cautiously because education may proxy reporting, recall, fishing practices or unmeasured socio-economic differences rather than a direct causal effect. See Table 4.

## 4. Discussion

This study shows that crocodile-related fishing-gear damage in the sampled southern Sudd communities is seasonal, socially reported through informal channels and perceived as difficult to resolve through formal institutions. The strongest empirical pattern was seasonality: quarterly ordinal scores increased sharply from the first half of the year to July–September and October–December, while September was the dominant single selected month. The study therefore adds an indirect livelihood dimension to the literature on human–crocodile conflict, which has often emphasized attacks on people and livestock [4,14,15,16].

The seasonal pattern is consistent with a plausible overlap between fishing activity, floodplain productivity and crocodile foraging and with previous evidence that Nile crocodile impacts can impose material costs on rural fishing and wetland livelihoods [50]. During the wet and floodplain periods, fishers may place nets in habitats where fish are abundant and where crocodiles may also forage, move or disperse. In such settings, crocodiles may depredate captured fish from nets, tear nets while feeding or moving through them, or damage gear when attracted to concentrated fish. Similar seasonal structuring of human–crocodile interactions has been reported in other systems where hydrology, fishing activity and crocodile habitat use change across the year [4,10,11,12]. The apparent discrepancy between the September monthly peak and high October–December quarterly scores is resolved by recognizing that the questions measured different temporal scales: one asked for a single main month, while the other scored frequency across a three-month period.

Broader-area differences were less clearly separated than seasonal differences in the fitted ordinal models. This does not mean that local heterogeneity was absent. Any observed differences among Terekeka, Northern Terekeka, Gemeiza, Tombek and Mangalla may reflect local hydrology, proximity of fishing locations to White Nile channels and floodplain margins, timing and intensity of fishing activity, type and number of nets used, net-setting locations, crocodile encounter rates and access to local reporting or management institutions. These mechanisms were not measured independently in the questionnaire, so they are treated as plausible explanatory factors rather than as directly tested causal predictors.

The reporting-pathway results demonstrate that local reporting does not necessarily mean formal wildlife reporting. All respondents indicated that incidents were communicated to other fishers, family members and fishing-camp leadership, whereas none reported wildlife authorities as a reporting channel. This suggests that community-based communication is active but formal wildlife-management systems are either inaccessible, distrusted or not perceived as useful for gear-damage incidents. Similar institutional dimensions are central to human–wildlife conflict elsewhere, where tolerance depends not only on the damage itself but also on whether communities believe authorities can respond fairly and practically [2,7,8,9]. The finding that 85% reported fisheries authorities but 0% reported wildlife authorities indicates a mismatch between where fishers seek help and where crocodile-management mandates may sit.

Conflict-resolution perceptions were especially important. Universal agreement that gear-damage conflicts are difficult to resolve, combined with no expectation of government compensation or Department of Wildlife resolution, points to low institutional confidence. This result should not be interpreted simply as fatalism. In remote wetland areas, formal agencies may be physically distant, under-resourced or perceived as unable to compensate for recurring gear losses. The high agreement that “only God can resolve” the conflict may reflect a combination of religious framing, resignation and perceived absence of effective institutional alternatives. Management responses should therefore prioritize trusted local reporting systems before assuming that formal reporting channels alone will capture the scale of gear damage.

Responses to damaged gear indicate direct livelihood consequences. Repair was rare, while abandonment and replacement were universal in the agreement responses. This suggests that crocodile damage may often make nets unusable or not worth repairing. For established fishers with multiple nets, replacement may be possible but costly; for new entrants with only a few nets, losing one or two nets could interrupt fishing, force borrowing, require wage labour or trigger shifts to other livelihoods. This pathway is consistent with broader human-dimensions work showing that coexistence costs are unevenly distributed and can be most severe for households with limited assets [5,9]. Future work should quantify replacement costs, lost fishing time and catch reduction directly because the present questionnaire measured damaged-net categories rather than monetary loss.

The regression results should be interpreted as support for patterns within sampled respondents rather than as population-level risk estimates. The quarterly ordinal model showed seasonal effects, and including respondent or location random intercepts did not change this conclusion. The annual damaged-net category varied by survey year, but year effects may reflect hydrological conditions, sampling distribution, local reporting, fishing effort or other unmeasured factors. The education association with the annual damaged-net category should be treated cautiously because education may be correlated with recall, interpretation of categories, gear ownership, fishing strategy or unmeasured socio-economic differences. The formal-reporting null model and the decision not to model outcomes with no variation or fewer than 10 affirmative events are also important: they prevent over-interpretation of models that the data cannot support.

The study has several limitations. First, sampling was purposive and focused on crocodile-exposed communities and respondents with relevant local experience. The findings therefore cannot estimate the overall prevalence of fishing-gear damage among all fishers in the Sudd Wetlands, the true spatial distribution of risk across the wetland or reporting behaviour across South Sudan. Second, the results are based on self-reported questionnaire responses and may be affected by recall bias, social desirability and differences in how respondents interpreted ordinal categories. Third, women were under-represented, and future research should include female interviewers and gender-sensitive sampling strategies to better capture women’s experience of wetland livelihoods and crocodile conflict. Fourth, the survey did not measure crocodile abundance, crocodile movement, fishing effort, gear value, catch loss or exact replacement costs. Fifth, location clustering was evaluated statistically, but some village/fishing locations had small sample sizes, limiting precise location-level inference.

Despite these limitations, the findings have practical implications. First, seasonal warnings and mitigation trials should focus on the July–December period, especially the August–September peak and the late wet-season quarter. Second, reporting systems should build on existing trusted channels, especially fishing-camp leadership and fisher-to-fisher communication, while creating a clear link to fisheries and wildlife authorities. Third, conflict-resolution mechanisms should acknowledge gear damage as a legitimate livelihood loss rather than focusing only on human and livestock attacks. Fourth, pilot interventions should test affordable net-protection, gear-repair, replacement-credit or compensation-record systems in collaboration with local fishing camps. Fifth, any compensation or support scheme should require standardized documentation of incidents because informal reports alone may not provide enough evidence for fair allocation.

## 5. Conclusions

Nile crocodile damage to fishing gear in the sampled southern Sudd communities was seasonal, concentrated in late wet-season months and communicated mainly through informal community reporting pathways. Respondents reported limited confidence in formal conflict-resolution or compensation systems, and damaged nets were generally abandoned and replaced rather than repaired. These findings support a management approach that combines seasonal risk communication, community-based incident documentation, stronger links between fisheries and wildlife authorities, and practical support for gear protection or replacement. Because the study used purposive sampling, conclusions apply to the sampled crocodile-exposed communities and should not be generalized as prevalence estimates for all fishers in the Sudd Wetlands or South Sudan.

## Figures and Tables

**Table 1:** Research questions, response variables and statistical analyses used in the study. Detailed candidate regression models and interpretation status are summarized separately in Table 3.

Question or Analytical Purpose	Response Variable	Main Analysis
Does reported damage vary seasonally?	Quarterly ordered fishing-gear damage frequency	Descriptive ordinal distributions, Friedman test and cumulative link models.
Which months were reported as the main damage period?	Selected main damage month	Monthly frequency table and chi-square goodness-of-fit test.
Are the quarterly and monthly seasonality variables consistent?	Selected main month crossed with quarterly damage response	Cross-tabulations used as data-checking and interpretation outputs.
How were gear-damage incidents communicated?	Agreement with incident-reporting channel items	Agreement percentages; formal institutional reporting analysed as a supplementary binary outcome.
How did respondents perceive conflict resolution?	Agreement with conflict-resolution statements	Agreement percentages.
What actions were taken after fishing gear was damaged?	Agreement with repair, abandonment, borrowing and purchase responses	Agreement percentages and Cochran’s Q test; borrowing analysed as a supplementary binary outcome.
What annual damaged-net category was most likely?	Mutually exclusive annual damaged-net category	Descriptive category distribution and proportional-odds ordinal logistic regression.
Was location structure considered?	Village/fishing location, broader area and coordinates	Location sample sizes, survey-location map and location random effects in quarterly ordinal models.
Were categorical predictors strongly associated?	Pairs of categorical predictors	Cramér’s *V* used as a descriptive association effect-size measure, not as a linear correlation coefficient.

**Table 2:** Sample profile of the analytical dataset.

Category	Level	Value
Total respondents	–	379
Broader areas	–	5
Villages/fishing locations	–	27
Survey years	–	2018–2020
Gender record	Male	357
Gender record	Female	21
Gender record	Not coded	1
Area	Terekeka	83 (21.9%)
Area	Tombek	81 (21.4%)
Area	Gemeiza	80 (21.1%)
Area	Mangalla	70 (18.5%)
Area	Northern Terekeka	65 (17.2%)
Age group	19–29 years	103
Age group	30–49 years	189
Age group	50–69 years	76
Age group	≥70 years	10

**Table 4:** Selected regression results from the main interpretable models. Odds ratios greater than one indicate higher odds of a higher ordered response category.

Outcome	Predictor	Ratio	95% CI	*p* Value
Quarterly damage intensity	Jan–Mar vs. Apr–Jun	OR 0.33	0.25–0.43	<0.001
Quarterly damage intensity	Jul–Sept vs. Apr–Jun	OR 24.51	17.55–34.23	<0.001
Quarterly damage intensity	Oct–Dec vs. Apr–Jun	OR 40.22	28.14–57.48	<0.001
Annual damaged-net category	Year 2019 vs. 2018	OR 0.31	0.17–0.56	<0.001
Annual damaged-net category	Year 2020 vs. 2018	OR 0.21	0.11–0.37	<0.001
Annual damaged-net category	University education	OR 4.86	1.63–14.47	0.004

Reference categories were April–June for quarterly models and 2018 for year effects. The university education term is relative to the reference education category in the coded dataset and is interpreted cautiously. Estimates are shown only as selected interpretable results from outcome-specific models; odds ratios should not be compared across different response variables.

## Data Availability

The anonymized analytical dataset and R analysis script can be made available by the corresponding author upon reasonable request, subject to ethical, community-consent and institutional constraints. The revised analysis script records the R-session information and package versions used to generate the results.

## Supplementary Materials

The following supporting information can be downloaded at: https://www.mdpi.com/article/10.3390/ani16182826/s1.

## Appendix A. Analysed Questionnaire Items and Coding

**Table A1:** Summary of analysed questionnaire components. The full questionnaire is submitted as Supplementary File S1.

Component	Question/Information Used in Analysis	Response Format/Coding	Main Analysis
Quarterly damage	Frequency of fishing-gear damage in Jan–Mar, Apr–Jun, Jul–Sept and Oct–Dec	Ordered categories coded 1 = never to 5 = most of the time	Friedman test; cumulative link mixed/fixed models
Main damage month	Main month reported for fishing-gear damage	Month selected from Jan–Dec	Frequency table; chi-square goodness-of-fit; cross-tabulation with quarterly response
Reporting pathways	Whether incidents were reported to other fishers, family, camp leadership, fisheries authorities, community leaders, research/academia or wildlife authorities	Agreement/strong agreement combined as agreement	Agreement percentages; formal-reporting logistic regression where applicable
Conflict resolution	Perceptions that conflict is difficult to resolve, can be resolved by the Department of Wildlife, compensated for by government, resolved only by God or reduced by stopping fishing in sensitive zones	Agreement/strong agreement combined as agreement	Agreement percentages
Responses to damaged gear	Repair damaged nets, abandon damaged nets, borrow nets from colleagues, purchase new nets	Agreement/strong agreement combined as agreement	Agreement percentages; Cochran’s Q; borrowing logistic regression where applicable
Annual damaged nets	Estimated annual number of damaged nets	1–5, 6–10, 11–15, >20 nets/year/participant; mutually exclusive likely category derived for main figure	Ordinal logistic regression; descriptive figure
Location and area	Village/fishing location, broader area, coordinates	Cleaned location labels; converted coordinates where possible	Location counts; location random effects; survey-location map
Respondent covariates	Survey year, gender, age group, education and origin status	Categorical predictors	Candidate regression covariates; Cramér’s *V* used as a descriptive association effect-size measure for categorical predictor pairs.

## References

[1] Mitsch W.J., Gosselink J.G. (2015). Wetlands.

[2] IUCN SSC Human-Wildlife Conflict Task Force (2020). IUCN SSC Position Statement on the Management of Human-Wildlife Conflict.

[3] Pooley S. (2016). The entangled relations of humans and Nile crocodiles in Africa, c. 1840–1992. Environ. Hist..

[4] Pooley S., Botha H., Combrink X., Powell G. (2020). Synthesizing Nile crocodile attack data and historical context to inform mitigation in southern Africa. Oryx.

[5] Cavalier R., Pratt E.N., Serenari C., Rubino E.C. (2022). Human dimensions of crocodilians: A review of the drivers of coexistence. Hum. Dimens. Wildl..

[6] Woodroffe R., Thirgood S., Rabinowitz A. (2005). People and Wildlife: Conflict or Coexistence?.

[7] Dickman A.J. (2010). Complexities of conflict: The importance of considering social factors for effectively resolving human–wildlife conflict. Anim. Conserv..

[8] Redpath S.M., Young J., Evely A., Adams W.M., Sutherland W.J., Whitehouse A., Amar A., Lambert R.A., Linnell J.D.C., Watt A. (2013). Understanding and managing conservation conflicts. Trends Ecol. Evol..

[9] Nyhus P.J. (2016). Human–wildlife conflict and coexistence. Annu. Rev. Environ. Resour..

[10] Das C.S., Jana R. (2018). Human–crocodile conflict in the Indian Sundarban: An analysis of spatio-temporal incidences in relation to people’s livelihood. Oryx.

[11] García-Grajales J., Buenrostro-Silva A. (2019). Assessment of human–crocodile conflict in Mexico: Patterns, trends and hotspot areas. Mar. Freshw. Res..

[12] Khan W., Hore U., Mukherjee S., Mallapur G. (2020). Human–crocodile conflict and attitude of local communities toward crocodile conservation in Bhitarkanika Wildlife Sanctuary, Odisha, India. Mar. Policy.

[13] Brien M.L., Gienger C.M., Browne C.A., Read M.A., Joyce M.J., Sullivan S. (2017). Patterns of human–crocodile conflict in Queensland: A review of historical estuarine crocodile (*Crocodylus porosus*) management. Wildl. Res..

[14] Dunham K.M., Ghiurghi A., Cumbi R., Urbano F. (2010). Human–wildlife conflict in Mozambique: A national perspective, with emphasis on wildlife attacks on humans. Oryx.

[15] Wallace K.M., Leslie A.J., Coulson T. (2011). Living with predators: A focus on the issues of human–crocodile conflict within the lower Zambezi Valley. Wildl. Res..

[16] Chihona S., Powell G., Pooley S. (2023). Negative human–crocodile interactions in Kariba, Zimbabwe: Data to support potential mitigation strategies. Oryx.

[17] Fergusson R., Manolis S.C., Stevenson C. (2010). Nile crocodile, *Crocodylus niloticus*. Crocodiles: Status Survey and Conservation Action Plan.

[18] Isberg S., Combrink X., Lippai C., Balaguera-Reina S.A. (2019). *Crocodylus* *niloticus*. Iucn Red List Threat. Species.

[19] Mohamed Y.A., van den Hurk B.J.J.M., Savenije H.H.G., Bastiaanssen W.G.M. (2005). Hydroclimatology of the Nile: Results from a regional climate model. Hydrol. Earth Syst. Sci..

[20] Petersen G., Abya J.A., Fohrer N. (2007). Spatio-temporal water body and vegetation changes in the Nile swamps of southern Sudan. Adv. Geosci..

[21] UNESCO World Heritage Centre (2017). The Sudd Wetland.

[22] Nile Basin Initiative (2022). Sudd Wetland Monograph, Volume 1: Overview of the Sudd Wetland and Land Use.

[23] Benansio J.S., Demaya G.S., Dendi D., Luiselli L. (2022). Attacks by Nile crocodiles (*Crocodylus niloticus*) on humans and livestock in the Sudd Wetlands, South Sudan. Russ. J. Herpetol..

[24] Benansio J.S., Dendi D., Fa J.E., Luiselli L. (2024). Attitudes and perceptions of local communities towards Nile crocodiles (*Crocodylus niloticus*) in the Sudd Wetlands, South Sudan. Animals.

[25] R Core Team (2025). R: A Language and Environment for Statistical Computing.

[26] Readr Developers *readr: Read Rectangular Text Data*; R Package; 2025. https://github.com/tidyverse/readr.

[27] Wickham H., François R., Henry L., Müller K., Vaughan D. *dplyr: A Grammar of Data Manipulation*; R Package; 2025. https://github.com/tidyverse/dplyr.

[28] Tidyr Developers *tidyr: Tidy Messy Data*; R Package; 2025. https://github.com/tidyverse/tidyr.

[29] Stringr Developers *stringr: Simple, Consistent Wrappers for Common String Operations*; R Package; 2025. https://github.com/tidyverse/stringr.

[30] Forcats Developers *forcats: Tools for Working with Categorical Variables*; R Package; 2025. https://github.com/tidyverse/forcats.

[31] Purrr Developers *purrr: Functional Programming Tools*; R Package; 2025. https://github.com/tidyverse/purrr.

[32] Wickham H. (2016). ggplot2: Elegant Graphics for Data Analysis.

[33] Scales Developers *scales: Scale Functions for Visualization*; R Package; 2025. https://r-lib.r-universe.dev/scales.

[34] Garnier S., Ross N., Rudis B., Sciaini M., Scherer C., Camargo A.P. *viridis: Colorblind-Friendly Color Maps for R*; R Package; 2025. https://cran.r-project.org/web/packages/viridis/index.html.

[35] Pedersen T.L. *patchwork: The Composer of Plots*; R Package; 2025. https://thomasp85.r-universe.dev/patchwork.

[36] Ragg developers *ragg: Graphic Devices Based on AGG*; R Package; 2025. https://ragg.r-lib.org/.

[37] Broom Developers *broom: Convert Statistical Objects into Tidy Tibbles*; R Package; 2025. https://github.com/tidyverse/broom.

[38] Christensen R.H.B. *ordinal: Regression Models for Ordinal Data*; R Package; 2025. https://cran.r-project.org/web/packages/ordinal/index.html.

[39] Venables W.N., Ripley B.D. (2002). Modern Applied Statistics with S.

[40] Agresti A. (2010). Analysis of Ordinal Categorical Data.

[41] McCullagh P. (1980). Regression models for ordinal data. J. R. Stat. Soc. Ser. B.

[42] Hosmer D.W., Lemeshow S., Sturdivant R.X. (2013). Applied Logistic Regression.

[43] McCullagh P., Nelder J.A. (1989). Generalized Linear Models.

[44] Hilbe J.M. (2011). Negative Binomial Regression.

[45] Burnham K.P., Anderson D.R. (2002). Model Selection and Multimodel Inference: A Practical Information-Theoretic Approach.

[46] Pebesma E. (2018). Simple features for R: Standardized support for spatial vector data. R J..

[47] Massicotte P., South A. *rnaturalearth: World Map Data from Natural Earth*; R Package; 2025. https://ropensci.r-universe.dev/rnaturalearth.

[48] South A., Michael S., Massicotte P. *rnaturalearthdata: World Vector Map Data from Natural Earth Used in rnaturalearth*; R Package; 2025. https://ropensci.r-universe.dev/rnaturalearthdata.

[49] Dunnington D. *ggspatial: Spatial Data Framework for ggplot2*; R Package; 2025. https://cran.r-project.org/web/packages/ggspatial/index.html.

[50] Aust P.W., Boyle B., Fergusson R., Coulson T. (2009). The impact of Nile crocodiles on rural livelihoods in northeastern Namibia. S. Afr. J. Wildl. Res..

