# Supplementary material for: Seasonal Nile Crocodile Damage to Fishing Gear, Incident Reporting and Livelihood Responses in the Sudd Wetlands, South Sudan
Source: Animals (Basel). 2026 Sep 8;16(18):2826. doi: 10.3390/ani16182826 (PMC13603671; doi:10.3390/ani16182826)
Supplement: Supplementary file 1 [file animals-16-02826-s001.zip › Supplementary-file S1.pdf]

CROCODILE –HUMAN CONFLICTS SURVEY IN SUDD WETLAND, SOUTH SUDAN.

1.	**SECTION I: DEMOGRAPHY**					
1.1.	Date	Location		Coordinate		
1.2.	Name of Respondent			Tel:		
2.2.	**Gender Status**					
	Male	Female				
	**Age of Respondent**					
1.3.	18-29 years	30-49 years	50-69 years	Above 70 years		
1.4.	**Marital status**					
	Single	Married	Divorced	Separate	Widow/Widower	
1.5.	**Educational Background**					
	No formal education	Primary level	Secondary level	Diploma	Vocational School	
	Bachelor degree	M.Sc. degree	Ph.D.	Other specified		
	Are you original from this area?					
	YES	NO				
	If your answer NO; where do you come from?					
2.	**SECTION II: DEMAGAE OF FISHING GEARS CAUSE BY NILE CROCODILES**					
2.1.	**How frequently do you experience the damages of fishing gears by Nile Crocodiles in this area?**					
		Most of the time	Often	Occasional	Rarely	Never
	First Quarter of the year (Jan-March)					
	Second Quarter of the year (April-June)					
	Third Quarter of the year (July-Sept)					
	Fourth Quarter of the year (Oct-Dec)					
2.2.	**How do the local fisher's community resolve Nile Crocodile Conflicts relate to damage of fishing gears in this area?**					
		Strongly agree	Agree	Unsure	Disagree	Strongly disagree
	Difficult to Resolve					
	Department of Wildlife to resolve					
	Government compensate the damage fishing gears					
	Only god can resolve					
	Stop fishing in Nile Crocodile sensitive zones					
	Other specify					
2.3.	**Do the local fishers community report the incidences of fishing gear damages cause by Nile**					

	**Crocodile to the government authorities in the area?**					
		Strongly agree	Agree	Unsure	Disagree	Strongly disagree
	Wildlife Authorities					
	Fisheries Authorities					
	Leaderships of the fishing camp					
	Family members					
	Other fishers					
	Community Leader					
	Research Institution & Academia					
	Other specify					
2.4.	**How do the local fishers handle damage fishing gears caused by Nile Crocodile in this area**					
		Strongly agree	Agree	Unsure	Disagree	Strongly disagree
	Repair the damage fishing nets					
	Abundant the damage fishing nets					
	Borrow fishing nets from colleagues					
	Purchase brand new fishing net					
	Other specify					
2.5.	**What is the main season local fishers experiences the damages of fishing gears caused by Nile Crocodiles in this area?**					

	Jan	Feb	March	April	May	Jun	Jul	Aug	Sept	Oct	Nov	Dec

2.6.	**What is the estimated numbers of your households fishing gears been damage by Nile Crocodiles in this area over the past 12 Months?**					
		Strongly agree	Agree	Unsure	Disagree	Strongly disagree
	1-5 fishing nets/year/fisher					
	6-10 fishing nets/year/fisher					
	11-15 fishing nets/year/fisher					
	>20 fishing nets/year/fisher					
	Other specify					
**3.**	**SECTION III: BY-CATCH OF JUVENILE NILE CROCODILE BY THE LOCAL FISHERS COMMUNITY**					
3.1.	**How frequently do the local fishers experience the incidence of Juvenile Nile Crocodile by-catch in this area**					

		Most of the time	Often	Occasional	Rarely	Never
	First Quarter of the Year (Jan -March)					
	Second Quarter of the Year (April -June)					
	Third Quarter of the Year (July-Sept)					
	Fourth Quarter of the Year (Oct- Dec)					

3.2. **Do the local fishers community report the incidence of Juvenile Nile by-catch to the government authorities in the area?**

		Strongly agree	Agree	Unsure	Disagree	Strongly disagree
	Wildlife Authorities					
	Fisheries Authorities					
	Leaderships of the fishing camp					
	Family members					
	Other fishers					
	Community Leaders					
	Other specify					

3.3. **How do the local fishers handle the juvenile Nile crocodile by-catch in this area?**

		Strongly agree	Agree	Unsure	Disagree	Strongly disagree
	Released juvenile crocodile back to the natural habitats					
	Sold juvenile crocodile to individual practicing farming					
	Killed juvenile crocodile immediately for local consumption					
	Killed juvenile crocodile to be used ornamental purpose					
	Killed and smoked juvenile crocodile for later consumption					
	Killed and smoked juvenile crocodile for marketing purpose					
	Other specify					

3.4. **What are the best season for local fishers to capture Juvenile Nile Crocodile by-catch in this area?**

Jan	Feb	March	April	May	Jun	Jul	Aug	Sept	Oct	Nov	Dec

What is the estimated number of Juvenile Nile Crocodile by-catch your households have capture in this area over the past 12 months?

		Strongly agree	Agree	Unsure	Disagree	Strongly disagree

	1-5 Juvenile Nile Crocodiles by-catch/year/fisher					
	6-10 Juvenile Nile Crocodiles by-catch/year/fisher					
	11-15 Juvenile Nile Crocodiles by-catch/year/fisher					
	16-20 Juvenile Nile Crocodiles by-catch/year/fisher					
	>25 Juvenile Nile Crocodiles by-catch/year/fisher					
**4.**	**SECTION IV: SIGHTING OF NILE CROCODILE BY THE LOCAL FISHERS COMMMUNITY**					
4.1.	**How frequently do the local fishers experience sight of Nile Crocodiles in this area?**					
		All year around	Most of the time	Sometimes	Rarely	Never
	First Quarter of the Year (Jan -March)					
	Second Quarter of the Year (April -June)					
	Third Quarter of the Year (July-Sept)					
	Fourth Quarter of the Year (Oct- Dec)					
4.2.	**What is the reaction of local fishers on sighting Nile Crocodile in this area?**					
		Strongly agree	Agree	Unsure	Disagree	Strongly disagree
	Flee the area					
	Map and Monitor the area					
	Retaliate to hunt Crocodile					
	Setup Crocodile trap in the area					
	Other specify					
4.3.	**How often do the local fishers communities sighted Nile Crocodile without conflict in this area?**					
		Most of the times	Occasional	Rarely	Never	
	First Quarter of the Year (Jan -March)					
	Second Quarter of the Year (April -June)					
	Third Quarter of the Year (July-Sept)					
	Fourth Quarter of the Year (Oct- Dec)					
4.4.	**What are the best season for sighting Nile Crocodiles in this area?**					

	Jan	Feb	March	April	May	Jun	Jul	Aug	Sept	Oct	Nov	Dec

4.5.	**How will the local community noticed that Nile Crocodile attack will occur the area?**					
		Strongly agree	Agree	Unsure	Disagree	Strongly disagree

	Sighting Crocodile by chance at the edge of River Bank					
	Extreme strong smell of fish-like in the area					
	Appearance of Crocodile Bird at the edge of River Bank					
	Jumping of Juvenile Fish on the surface of water					
	Slow movement of grass-patches opposite water current					
	Other specify					
**5.**	**SECTION V: LOCAL KNOWLEDGE ON REPRODUCTIVE BIOLOGY OF NILE CROCODILE**					
5.1.	**What is the main habitats Nile Crocodiles breeding in this area?**					
		Strongly agree	Agree	Unsure	Disagree	Strongly disagree
	Sandy area surrounding the river					
	Muddy area surrounding the river					
	Clay area surrounding the river					
	Sandy and clay area surrounding the river					
	Vegetation area surrounding the river					
	Deep water area of the river					
	Small creek associated with river					
	Other specify					
5.2.	**What is the main season for Nile Crocodiles reproductive biology in this area?**					

	Jan	Feb	March	April	May	Jun	Jul	Aug	Sept	Oct	Nov	Dec

5.3.	**How long does it take for Nile Crocodiles to hatch their eggs in the natural habitat?**					
		Strongly agree	Agree	Unsure	Disagree	Strongly disagree
	30 days for eggs to hatch					
	45 days for eggs to hatch					
	60 days for eggs to hatch					
	75 days for eggs to hatch					
	90 days for egg to hatch					
	120 days for eggs to hatch					
	Other specify					
5.4.	**what are the major threats/challenges affecting the nesting biology of Nile Crocodiles in this areas/villages**					
		Strongly agree	Agree	Unsure	Disagree	Strongly disagree
	Predation by monitor lizard					
	Habitat degradation					

	Predation by birds					
	Predation by hyena					
	Climate change					
	Hunting of nesting female					
	Gathering of nesting eggs for local consumption					
	Monitoring of nesting eggs site by local community					
	other specify					

Thanks you very much for your participation
