# Supplementary material for: Seasonal Nile Crocodile Damage to Fishing Gear, Incident Reporting and Livelihood Responses in the Sudd Wetlands, South Sudan
Source: Animals (Basel). 2026 Sep 8;16(18):2826. doi: 10.3390/ani16182826 (PMC13603671; doi:10.3390/ani16182826)
Supplement: Supplementary file 1 [file animals-16-02826-s001.zip › Supplementary_fileS2.pdf]

Supplementary file

Seasonality, reporting pathways and livelihood responses to Nile crocodile damage to fishing gear in the Sudd Wetlands, South Sudan

This file contains the supplementary tables and supporting statistical outputs referenced in the revised manuscript, excluding the full questionnaire / Supplementary File S1.

Supplementary Table S1. Respondent distribution by location

A: Respondents by village or fishing location.

Village/fishing location	n	Percent of analytical sample
Tombek	81	21.400
Goitabet/Lojelet	28	7.400
Lugana/Lubara	23	6.100
Juba-Bayalk	22	5.800
Leggeri/Lagari	21	5.500
Terekeka	18	4.700
Kworijik	16	4.200
Bore/Bori	14	3.700
Momonu	14	3.700
Jay	12	3.200
Kokot	12	3.200
Lwoki	12	3.200
Sernum	11	2.900
Bangosoro	9	2.400
Jeleri	9	2.400
Karalele	9	2.400
Nyori	9	2.400
Pakuor	8	2.100
Kejo-Keji	7	1.800
Nyokanita	7	1.800
Anyara	6	1.600
Lolifiong	6	1.600
Tibari	6	1.600
Karous	5	1.300
Palek	5	1.300
Sudan-Safari	5	1.300
Gemeiza	4	1.100

Percentages are based on the analytical dataset of 379 respondents.

B: Respondents by broader area and village/fishing location.

Area	Village/fishing location	n	Percent within area
Gemeiza	Jay	12	15.000
Gemeiza	Karalele	9	11.200
Gemeiza	Pakuor	8	10.000
Gemeiza	Kejo-Keji	7	8.800
Gemeiza	Nyokanita	7	8.800
Gemeiza	Anyara	6	7.500
Gemeiza	Lolifiong	6	7.500
Gemeiza	Tibari	6	7.500

Continued on next page

B: Respondents by broader area and village/fishing location. (continued)

Area	Village/fishing location	n	Percent within area
Gemeiza	Karous	5	6.200
Gemeiza	Palek	5	6.200
Gemeiza	Sudan-Safari	5	6.200
Gemeiza	Gemeiza	4	5.000
Mangalla	Leggeri/Lagari	21	30.000
Mangalla	Kworijik	16	22.900
Mangalla	Kokot	12	17.100
Mangalla	Lwoki	12	17.100
Mangalla	Nyori	9	12.900
Northern Terekeka	Goitabet/Lojelet	28	43.100
Northern Terekeka	Lugana/Lubara	23	35.400
Northern Terekeka	Bore/Bori	14	21.500
Terekeka	Juba-Bayalk	22	26.500
Terekeka	Terekeka	18	21.700
Terekeka	Momonu	14	16.900
Terekeka	Sernum	11	13.300
Terekeka	Bangosoro	9	10.800
Terekeka	Jeleri	9	10.800
Tombek	Tombek	81	100.000

This table provides the area-level structure underlying the 27 location counts.

Supplementary Table S2. Cross-tabulations of quarterly damage responses and selected main damage month

A: Quarterly damage response by selected main damage-month quarter.

Quarterly question	Quarterly response	Selected main-month quarter	n	Percent within quarter-response
Jan-Mar	Most of the time	Apr-Jun	3	17.600
Jan-Mar	Most of the time	Jul-Sept	10	58.800
Jan-Mar	Most of the time	Oct-Dec	4	23.500
Jan-Mar	Often	Jul-Sept	13	100.000
Jan-Mar	Occasionally	Apr-Jun	3	4.800
Jan-Mar	Occasionally	Jul-Sept	55	88.700
Jan-Mar	Occasionally	Oct-Dec	4	6.500
Jan-Mar	Rarely	Apr-Jun	10	10.400
Jan-Mar	Rarely	Jul-Sept	82	85.400
Jan-Mar	Rarely	Oct-Dec	4	4.200
Jan-Mar	Never	Apr-Jun	15	7.900
Jan-Mar	Never	Jul-Sept	158	82.700
Jan-Mar	Never	Oct-Dec	18	9.400
Apr-Jun	Most of the time	Apr-Jun	10	14.700
Apr-Jun	Most of the time	Jul-Sept	52	76.500
Apr-Jun	Most of the time	Oct-Dec	6	8.800
Apr-Jun	Often	Jul-Sept	55	98.200
Apr-Jun	Often	Oct-Dec	1	1.800
Apr-Jun	Occasionally	Apr-Jun	4	6.200
Apr-Jun	Occasionally	Jul-Sept	56	86.200
Apr-Jun	Occasionally	Oct-Dec	5	7.700
Apr-Jun	Rarely	Apr-Jun	4	5.700
Apr-Jun	Rarely	Jul-Sept	56	80.000

Continued on next page

A: Quarterly damage response by selected main damage-month quarter. (continued)

Quarterly question	Quarterly response	Selected main-month quarter	n	Percent within quarter-response
Apr-Jun	Rarely	Oct-Dec	10	14.300
Apr-Jun	Never	Apr-Jun	13	10.800
Apr-Jun	Never	Jul-Sept	99	82.500
Apr-Jun	Never	Oct-Dec	8	6.700
Jul-Sept	Most of the time	Apr-Jun	17	6.600
Jul-Sept	Most of the time	Jul-Sept	227	88.300
Jul-Sept	Most of the time	Oct-Dec	13	5.100
Jul-Sept	Often	Apr-Jun	13	11.700
Jul-Sept	Often	Jul-Sept	82	73.900
Jul-Sept	Often	Oct-Dec	16	14.400
Jul-Sept	Occasionally	Jul-Sept	8	100.000
Jul-Sept	Rarely	Oct-Dec	1	100.000
Jul-Sept	Never	Apr-Jun	1	50.000
Jul-Sept	Never	Jul-Sept	1	50.000
Oct-Dec	Most of the time	Apr-Jun	20	6.800
Oct-Dec	Most of the time	Jul-Sept	247	84.300
Oct-Dec	Most of the time	Oct-Dec	26	8.900
Oct-Dec	Often	Apr-Jun	11	12.900
Oct-Dec	Often	Jul-Sept	70	82.400
Oct-Dec	Often	Oct-Dec	4	4.700
Oct-Dec	Occasionally	Jul-Sept	1	100.000

This data-checking cross-tabulation shows that the monthly and quarterly variables measured different temporal scales.

B: Quarterly damage response by selected main damage month.

Quarterly question	Quarterly response	Selected month	Selected month quarter	n	Percent within quarter-response
Jan-Mar	Most of the time	Jun	Apr-Jun	3	17.600
Jan-Mar	Most of the time	Jul	Jul-Sept	2	11.800
Jan-Mar	Most of the time	Aug	Jul-Sept	2	11.800
Jan-Mar	Most of the time	Sept	Jul-Sept	6	35.300
Jan-Mar	Most of the time	Oct	Oct-Dec	4	23.500
Jan-Mar	Often	Jul	Jul-Sept	1	7.700
Jan-Mar	Often	Aug	Jul-Sept	4	30.800
Jan-Mar	Often	Sept	Jul-Sept	8	61.500
Jan-Mar	Occasionally	Jun	Apr-Jun	3	4.800
Jan-Mar	Occasionally	Jul	Jul-Sept	14	22.600
Jan-Mar	Occasionally	Aug	Jul-Sept	16	25.800
Jan-Mar	Occasionally	Sept	Jul-Sept	25	40.300
Jan-Mar	Occasionally	Oct	Oct-Dec	4	6.500
Jan-Mar	Rarely	Jun	Apr-Jun	10	10.400
Jan-Mar	Rarely	Jul	Jul-Sept	16	16.700
Jan-Mar	Rarely	Aug	Jul-Sept	29	30.200
Jan-Mar	Rarely	Sept	Jul-Sept	37	38.500
Jan-Mar	Rarely	Oct	Oct-Dec	4	4.200
Jan-Mar	Never	May	Apr-Jun	1	0.500
Jan-Mar	Never	Jun	Apr-Jun	14	7.300
Jan-Mar	Never	Jul	Jul-Sept	29	15.200
Jan-Mar	Never	Aug	Jul-Sept	48	25.100
Jan-Mar	Never	Sept	Jul-Sept	81	42.400
Jan-Mar	Never	Oct	Oct-Dec	18	9.400
Apr-Jun	Most of the time	Jun	Apr-Jun	10	14.700
Apr-Jun	Most of the time	Jul	Jul-Sept	13	19.100
Apr-Jun	Most of the time	Aug	Jul-Sept	13	19.100
Apr-Jun	Most of the time	Sept	Jul-Sept	26	38.200
Apr-Jun	Most of the time	Oct	Oct-Dec	6	8.800
Apr-Jun	Often	Jul	Jul-Sept	16	28.600
Apr-Jun	Often	Aug	Jul-Sept	17	30.400
Apr-Jun	Often	Sept	Jul-Sept	22	39.300
Apr-Jun	Often	Oct	Oct-Dec	1	1.800
Apr-Jun	Occasionally	Jun	Apr-Jun	4	6.200
Apr-Jun	Occasionally	Jul	Jul-Sept	8	12.300
Apr-Jun	Occasionally	Aug	Jul-Sept	22	33.800
Apr-Jun	Occasionally	Sept	Jul-Sept	26	40.000
Apr-Jun	Occasionally	Oct	Oct-Dec	5	7.700

Continued on next page

B: Quarterly damage response by selected main damage month. (continued)

Quarterly question	Quarterly response	Selected month	Selected month quarter	n	Percent within quarter-response
Apr-Jun	Rarely	May	Apr-Jun	1	1.400
Apr-Jun	Rarely	Jun	Apr-Jun	3	4.300
Apr-Jun	Rarely	Jul	Jul-Sept	8	11.400
Apr-Jun	Rarely	Aug	Jul-Sept	20	28.600
Apr-Jun	Rarely	Sept	Jul-Sept	28	40.000
Apr-Jun	Rarely	Oct	Oct-Dec	10	14.300
Apr-Jun	Never	Jun	Apr-Jun	13	10.800
Apr-Jun	Never	Jul	Jul-Sept	17	14.200
Apr-Jun	Never	Aug	Jul-Sept	27	22.500
Apr-Jun	Never	Sept	Jul-Sept	55	45.800
Apr-Jun	Never	Oct	Oct-Dec	8	6.700
Jul-Sept	Most of the time	May	Apr-Jun	1	0.400
Jul-Sept	Most of the time	Jun	Apr-Jun	16	6.200
Jul-Sept	Most of the time	Jul	Jul-Sept	43	16.700
Jul-Sept	Most of the time	Aug	Jul-Sept	68	26.500
Jul-Sept	Most of the time	Sept	Jul-Sept	116	45.100
Jul-Sept	Most of the time	Oct	Oct-Dec	13	5.100
Jul-Sept	Often	Jun	Apr-Jun	13	11.700
Jul-Sept	Often	Jul	Jul-Sept	17	15.300
Jul-Sept	Often	Aug	Jul-Sept	27	24.300
Jul-Sept	Often	Sept	Jul-Sept	38	34.200
Jul-Sept	Often	Oct	Oct-Dec	16	14.400
Jul-Sept	Occasionally	Jul	Jul-Sept	2	25.000
Jul-Sept	Occasionally	Aug	Jul-Sept	3	37.500
Jul-Sept	Occasionally	Sept	Jul-Sept	3	37.500
Jul-Sept	Rarely	Oct	Oct-Dec	1	100.000
Jul-Sept	Never	Jun	Apr-Jun	1	50.000
Jul-Sept	Never	Aug	Jul-Sept	1	50.000
Oct-Dec	Most of the time	May	Apr-Jun	1	0.300
Oct-Dec	Most of the time	Jun	Apr-Jun	19	6.500
Oct-Dec	Most of the time	Jul	Jul-Sept	49	16.700
Oct-Dec	Most of the time	Aug	Jul-Sept	77	26.300
Oct-Dec	Most of the time	Sept	Jul-Sept	121	41.300
Oct-Dec	Most of the time	Oct	Oct-Dec	26	8.900
Oct-Dec	Often	Jun	Apr-Jun	11	12.900
Oct-Dec	Often	Jul	Jul-Sept	13	15.300
Oct-Dec	Often	Aug	Jul-Sept	22	25.900
Oct-Dec	Often	Sept	Jul-Sept	35	41.200

Continued on next page

B: Quarterly damage response by selected main damage month. (continued)

Quarterly question	Quarterly response	Selected month	Selected month quarter	n	Percent within quarter-response
Oct-Dec	Often	Oct	Oct-Dec	4	4.700
Oct-Dec	Occasionally	Sept	Jul-Sept	1	100.000

Percentages are calculated within each quarterly response category.

Supplementary Table S3. Formal reporting logistic regression candidate set

A: Formal-reporting binary logistic model selection.

Model	AIC	BIC	logLik	df	ΔAIC
null	312.74	316.65	-155.37	1	0
area_year	317.40	344.77	-151.70	7	4.65
demographics	317.96	353.16	-149.98	9	5.22
full	323.58	382.24	-146.79	15	10.84

The null model had the lowest AIC; therefore, the main manuscript emphasizes descriptive reporting-channel results.

B: Formal-reporting null-model odds ratio output.

Term	Estimate	SE	z	P value	OR	CI low	CI high
(Intercept)	1.74	0.15	11.92	<0.001	5.71	4.29	7.60

Supplementary Table S4. Borrowing damaged-gear response model

A: Borrowing-net binary logistic model selection.

Model	AIC	BIC	logLik	df	ΔAIC
area_year	335.13	362.51	-160.57	7	0
full	337.66	396.32	-153.83	15	2.53
null	345.54	349.46	-171.77	1	10.41
demographics	350.54	385.74	-166.27	9	15.41

This model is supplementary because area and year effects have limited behavioural interpretation for individual borrowing decisions.

B: Odds ratios from the best-supported borrowing-net logistic model.

Term	OR	CI low	CI high	P value
(Intercept)	0.48	0.24	0.94	0.033
areaMangalla	1.63	0.73	3.62	0.230
areaNorthern Terekeka	0.42	0.15	1.18	0.099
areaTerekeka	0.67	0.29	1.57	0.356
areaTombek	0.73	0.32	1.69	0.465
year2019	0.49	0.25	0.97	0.040
year2020	0.30	0.15	0.58	<0.001

Supplementary Table S5. Annual damaged-net count sensitivity analysis

A: Poisson overdispersion check for approximate annual damaged-net count.

Best Poisson candidate	Overdispersion ratio
poisson_full	1.50

The count response used midpoint approximation of annual damaged-net categories and is interpreted only as a sensitivity analysis.

B: Poisson and negative-binomial model selection for approximate annual damaged-net count.

Model	AIC	BIC	logLik	df	ΔAIC
negbin_full	1662.65	1725.23	-815.33	16	0
negbin_area_year	1667.66	1698.95	-825.83	8	5.01
poisson_full	1688.82	1747.48	-829.41	15	26.16
negbin_null	1689.99	1697.81	-843.00	2	27.34
negbin_demographics	1691.37	1730.47	-835.68	10	28.71
poisson_area_year	1703.13	1730.50	-844.56	7	40.47
poisson_demographics	1736.12	1771.32	-859.06	9	73.47
poisson_null	1742.68	1746.59	-870.34	1	80.03

C: Rate ratios from the best-supported negative-binomial annual damaged-net count model.

Term	Rate ratio	CI low	CI high	P value
(Intercept)	6.01	4.93	7.32	<0.001
areaMangalla	0.87	0.72	1.05	0.138
areaNorthern Terekeka	0.99	0.82	1.19	0.899
areaTerekeka	0.89	0.75	1.06	0.198
areaTombek	1.05	0.88	1.25	0.582
year2019	0.72	0.62	0.83	<0.001
year2020	0.67	0.59	0.77	<0.001
gender2	0.74	0.56	0.98	0.035
age_group2	0.91	0.81	1.04	0.168
age_group3	1.00	0.83	1.19	0.956
age_group4	1.21	0.87	1.67	0.259
education2	1.07	0.92	1.24	0.401
education3	1.11	0.95	1.29	0.189
education4	1.53	1.18	1.99	0.001
origin_status2	0.85	0.61	1.19	0.343

Supplementary Table S6. Predictor-association screening

A: Pairwise Cramer's V among categorical candidate predictors.

Variable 1	Variable 2	Cramer's V
area	year	0.09
area	gender	0.10
area	age_group	0.11
area	education	0.22
area	origin_status	0.09
year	gender	0.08
year	age_group	0.08

Continued on next page

A: Pairwise Cramer's V among categorical candidate predictors. (continued)

Variable 1	Variable 2	Cramer's V
year	education	0.12
year	origin_status	0.11
gender	age_group	0.09
gender	education	0.15
gender	origin_status	0.02
age_group	education	0.12
age_group	origin_status	0.31
education	origin_status	0.10

No pairwise association was high enough to require exclusion of predictors on collinearity grounds.

Supplementary Table S7. Model-status log

A: Model-status log for the revised analysis.

Analysis section	Status	Message
Quarterly ordinal mixed regression	completed	Best quarterly mixed ordinal model by AIC: quarter_area_location_RE. Fixed-effect ordinal coefficient table reported from: quarter_area; rows: 1508; unique locations: 27.
Overall damage ordinal logistic regression	removed	Removed from the revised analysis: averaging ordinal quarterly responses was not retained. Quarterly cumulative link models are the primary damage-intensity regressions.
Formal reporting binary logistic regression	completed	Best binary model by AIC: null; complete cases: 369
Handling strategy binary logistic regression: repair	skipped	Binary model skipped: sparse outcome class; counts = 0:363, 1:6. Report descriptively or use a specialist rare-event/Firth logistic model.
Handling strategy binary logistic regression: abandon	skipped	Binary model skipped: fewer than 30 complete cases or only one outcome level.
Handling strategy binary logistic regression: borrow	completed	Best binary model by AIC: area_year; complete cases: 369
Handling strategy binary logistic regression: purchase	skipped	Binary model skipped: fewer than 30 complete cases or only one outcome level.
Annual gear-loss ordinal logistic regression	completed	Best ordinal logistic model by AIC: full; complete cases: 369
Annual gear-loss count regression	completed	Best count model by AIC: negbin_full; complete cases: 369

This table documents which models were retained, treated as descriptive only or skipped because outcome variation was insufficient.
